# The relationship between physical pain and suicidal thoughts and behaviors in adolescents: A meta-analysis

**DOI:** 10.1192/j.eurpsy.2021.1548

**Published:** 2021-08-13

**Authors:** M. De Filippi, M. Rignanese, E. Salmè, F. Madeddu, R. Calati

**Affiliations:** 1 Psychology, University of Milano-Bicocca, Milan, Italy; 2 Adult Psychiatry, Nîmes University Hospital, Nîmes, France

**Keywords:** Suicide, Suicidal Thoughts and Behaviors, adolescents, Physical Pain

## Abstract

**Introduction:**

Suicide is the third leading cause of death in adolescents (15-19 years). Physical pain is an important risk factor for suicidal thoughts and behaviors, especially in a delicate phase as adolescence.

**Objectives:**

Several studies investigated this association and the aim of this meta-analysis was to synthesize data in literature about this topic in adolescents (11-20 years).

**Methods:**

We started from a systematic review published by Hinze and colleagues in 2019 and we searched on PubMed (January 2019-June 2020) studies comparing rates of suicidal outcomes (Suicidal Ideation, Suicide Plan, Attempt, Self-Harm: SI, SP, SA, SH) in individuals with any type of physical pain (head, back, neck, chest, stomach, abdomen, muscle, joint, arthritis) vs. those without it. Data were analyzed with Comprehensive Meta-Analysis software (CMA, version 3).

**Results:**

Of the 16 included studies, eleven focused on SI (68.8%), six (37.5%) on SA, four (25%) on SH and two (12.5%) on SP. Adolescents with physical pain were more likely to report SI (p < .001), SH (p < .001), SA (p = .004) and SP (p = .006). In all analyses, the between study heterogeneity was high. The presence of publication bias has been detected in SI (k≥10).

**Conclusions:**

Results are in line with previous literature on this topic. Future research should investigate the specific impact of: acute vs. chronic pain; different types and intensities of pain; planned vs. impulsive action and therefore suicidal intent; role of psychological factors (in particular sensitivity and tolerance to physical pain).

